# Sex Difference in the Associations among Obesity-Related Indices with Metabolic Syndrome in Patients with Type 2 Diabetes Mellitus

**DOI:** 10.7150/ijms.63180

**Published:** 2021-08-13

**Authors:** Hsiu-Fen Jao, Chih-Hsuan Wung, Hui-Chen Yu, Mei-Yueh Lee, Po-Chih Chen, Szu-Chia Chen, Jer-Ming Chang

**Affiliations:** 1 Department of Laboratory Medicine, Kaohsiung Medical University Hospital, Kaohsiung Medical University, Kaohsiung, Taiwan.; 2 Department of post baccalaureate medicine, Kaohsiung Medical University, Kaohsiung, Taiwan.; 3 Department of Medical Imaging, Kaohsiung Medical University Hospital, Kaohsiung Medical University, Kaohsiung, Taiwan.; 4 Division of Endocrinology and Metabolism, Department of Internal Medicine, Kaohsiung Medical University Hospital, Kaohsiung Medical University, Kaohsiung, Taiwan.; 5 Department of Medical Laboratory Science and Biotechnology, College of Health Sciences, Kaohsiung Medical University, Kaohsiung, Taiwan.; 6 Department of Internal Medicine, Kaohsiung Municipal Siaogang Hospital, Kaohsiung Medical University, Kaohsiung, Taiwan.; 7 Division of Nephrology, Department of Internal Medicine, Kaohsiung Medical University Hospital, Kaohsiung Medical University, Kaohsiung, Taiwan.; 8 Faculty of Medicine, College of Medicine, Kaohsiung Medical University, Kaohsiung, Taiwan.

**Keywords:** sex difference, obesity-related indices, metabolic syndrome, diabetes mellitus

## Abstract

**Background:** The aim of this study was to investigate the associations among obesity-related indices and MetS in diabetic patients, and explore sex differences in these associations.

**Methods:** Patients with type 2 DM were included from two hospitals in southern Taiwan. The Adult Treatment Panel III criteria for an Asian population were used to define MetS. In addition, the following obesity-related indices were evaluated: waist-to-height ratio, waist-hip ratio (WHR), conicity index (CI), body mass index (BMI), body roundness index, body adiposity index, lipid accumulation product (LAP), abdominal volume index, visceral adiposity index (VAI), abdominal volume index and triglyceride-glucose index.

**Results:** A total of 1,872 patients with type 2 DM (mean age 64.0 ± 11.3 years, 808 males and 1,064 females) were enrolled. The prevalence rates of MetS were 59.8% and 76.4% in the males and female (*p* < 0.001), respectively. All of the obesity-related indices were associated with MetS in both sex (all *p* < 0.001). LAP and BMI had the greatest areas under the receiver operating characteristic curves in both sex. In addition, the interactions between BMI and sex (*p* = 0.036), WHR and sex (*p* = 0.016), and CI and sex (*p* = 0.026) on MetS were statistically significant.

**Conclusions:** In conclusion, this study demonstrated significant relationships between obesity-related indices and MetS among patients with type 2 DM. LAP and VAI were powerful predictors in both sex. The associations of BMI, WHR and CI on MetS were more significant in the men than in the women.

## Introduction

Metabolic syndrome (MetS) is a cluster of metabolic abnormalities including hypertension, insulin resistance (IR) and dyslipidemia 1, with an estimated global prevalence of around 25% 2. In Taiwan, the age-standardized prevalence according to the modified criteria for Asians is 16.4% 3. MetS has been reported to double the risk of atherosclerotic cardiovascular disease and increase the risk of type 2 diabetes mellitus (DM) by five-fold in patients without diabetes 4. In addition, prospective observational studies have demonstrated a strong association between MetS and the incidence of type 2 DM 5. In China and Ghana, 72.5% and 55.9% of patients with type 2 DM have been reported to have MetS, respectively 6, 7. Patients with the presence of both type 2 DM and MetS have been reported to be at a significant risk of developing cardiovascular complications 8. Moreover, MetS may be involved in the pathogenesis of both macro- and microvascular complications of DM 9.

Anthropometric indices such as waist-to-height ratio (WHtR), waist-hip ratio (WHR), conicity index (CI), body mass index (BMI), body roundness index (BRI), body adiposity index (BAI), lipid accumulation product (LAP), abdominal volume index (ABSI), visceral adiposity index (VAI), abdominal volume index (AVI) and triglyceride-glucose index (TyG) can easily be calculated and quantified using factors such as triglycerides (TGs), body height (BH), hip circumference (HC), waist circumference (WC) and body weight (BW) 10. These indices have been reported to be effective indicators of MetS in non-overweight/obese adults, vegetarians and in Nigeria 11-14. We recently identified associations between these obesity-related indices and nonalcoholic fatty liver disease and heavy metals 15, 16. However, few studies have investigated sex differences in the relationships among MetS and these obesity-related indices in patients with diabetes. Therefore, in this study, we collected the data of more than 1,800 patients with DM in southern Taiwan to investigate the associations among obesity-related indices and MetS, and further explored sex differences in these associations.

## Materials and methods

### Study Patients

**Figure 1:**
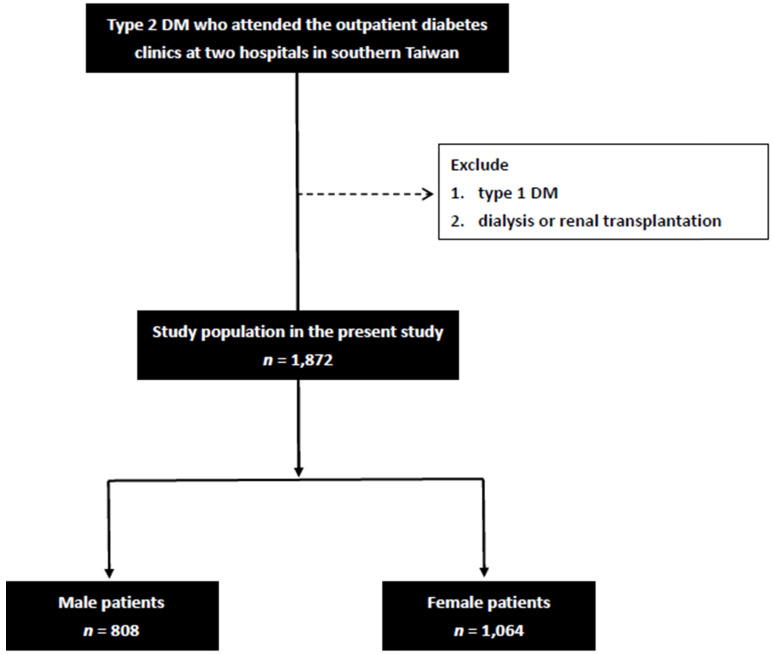
Flowchart of study population.

All patients with type 2 DM who attended the outpatient diabetes clinics at two hospitals in southern Taiwan were included in this study. The exclusion criteria were patients who: 1) had type 1 DM (defined as the presence of diabetic ketoacidosis, symptoms of severe hyperglycemia and ketonuria [≥3], or the continuous need for insulin in the year after the diagnosis), and 2) were receiving dialysis or had undergone a renal transplantation. Finally, 1,872 patients (mean age 64.0 ± 11.3 years, 808 males and 1,064 females) were included in this study (Figure 1). All of the included patients provided written informed consent to participate in this study, and the study protocol was approved by the Institutional Review Board of Kaohsiung Medical University Hospital. In addition, this study was conducted in accordance with the approved guidelines.

### Collection of demographic, medical, and laboratory data

All of the patients were interviewed, and their demographic and medical data including age, sex, and co-morbidities were recorded during these interviews and from medical records. Fasting blood samples were obtained from all of the patients, and laboratory tests were conducted using an autoanalyzer (Roche Diagnostics GmbH, D-68298 Mannheim COBAS Integra 400) for fasting glucose, TGs, total cholesterol, high-density lipoprotein cholesterol [HDL-C], low-density lipoprotein cholesterol [LDL-C] and estimated glomerular filtration rate (eGFR). Serum levels of creatinine were calculated using the compensated Jaffé (kinetic alkaline picrate) method using a calibrator that could traced in isotope-dilution mass spectrometry 17. EGFR values were calculated using the Chronic Kidney Disease Epidemiology Collaboration equation (CKD-EPI eGFR) 18. In addition, information on the use of the following medications during the study period was also obtained from medical records: insulin, oral anti-diabetic drugs, angiotensin II receptor blockers (ARBs), angiotensin converting enzyme inhibitors (ACEIs), fibrates and statins.

### Definition of MetS

The presence of MetS was defined according to the National Cholesterol Education Program Adult Treatment Panel (NCEP‑ATP) III guidelines 19 and modified criteria for Asians 20 as having three of the following five criteria: (1) elevated blood pressure (systolic blood pressure ≥ 130 mmHg, diastolic blood pressure ≥ 85 mmHg), a diagnosis of hypertension, or receiving treatment for hypertension; (2) hyperglycemia (fasting plasma glucose level ≥ 110 mg/dL or having been diagnosed with diabetes); (3) hypertriglyceridemia (TG concentration ≥ 150 mg/dL); (4) low concentration of HDL-C (< 40 mg/dL in men and < 50 mg/dL in women); and (5) abdominal obesity (WC > 90 cm in men and > 80 cm in women).

### Calculation of obesity-related indices

- BMI was calculated as:

BMI = BW (kg)/BH^2^ (m);

- WHR was calculated as:

WHR = WC (cm)/HC (cm);

- WHtR was calculated as:

WHtR = WC (cm)/BH (cm);

- LAP was calculated as:

LAP =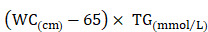 in males, and

LAP = 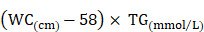 in females [21]

- BRI was calculated as:

BRI = 
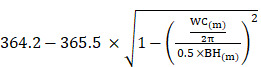
[22]

- CI was calculated using the Valdez equation based on BW, BH and WC as:

CI = 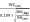
[23]

- VAI was calculated as described previously 24 using the following sex-specific equations (with TG levels in mmol/l and HDL-C levels in mmol/l):

VAI = 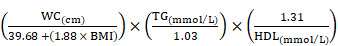 in males, and;

VAI = 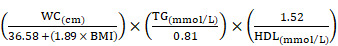 in females

- BAI was calculated according to the method of Bergman and colleagues as:

BAI = 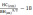
[25]

- AVI was calculated as:

AVI = 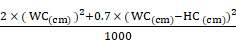
[26]

- ABSI was calculated as:

ABSI = WC (m)/ [BMI^2/3^(kg/m^2^) × BH^1/2^(m)] [27]

- TyG index was calculated as:

TyG index = Ln [fasting TG (mg/dL) × fasting plasma glucose (mg/dL)/2] [28]

### Statistical analysis

Statistical analysis was performed using SPSS 26.0 for Windows (SPSS Inc. Chicago, USA). Data are presented as number (percentage), mean (± standard deviation), or median (25^th^-75^th^ percentile) for TGs. Differences between groups were analyzed using the independent t-test for continuous variables and chi-square test for categorical variables. Multiple logistic regression analysis was used to identify factors associated with MetS. Covariates in the multivariable model included age, systolic and diastolic blood pressures, total cholesterol, LDL-C, eGFR and medications use. An interaction *p* in Logistic analysis: Model disease (y)= x1 +x2 + x1*x2 +covariates x1*x2 was interaction term. In this study, *y* = MetS; *x1* = sex; *x2* = each obesity-related index; covariates = age, systolic and diastolic blood pressures, total cholesterol, LDL-C, eGFR and medications use. Receiver operating characteristic (ROC) curves and areas under the ROC curves (AUCs) were used to assess the performance and predictive ability, respectively, of the obesity-related indices in identifying MetS. A difference was considered significant if the *p* value was less than 0.05.

## Results

A total of 1,872 patients were enrolled in this study. The mean age of the patients was 64.0 ± 11.3 years, and there were 808 males and 1,064 females. The prevalence rates of MetS were 59.8% and 76.4% in the males and females (*p* < 0.001), respectively.

### Comparison of baseline characteristics between the patients with and without MetS

The clinical characteristics of the patients with and without MetS and by sex are compared in Table 1. Compared to the male patients without MetS, the male patients with MetS were older, had higher systolic and diastolic blood pressures, higher BH, BW, WC, HC, and TG level, and lower HDL-C and eGFR. In addition, they had higher use rates of medications including oral anti-diabetic drugs, ACEIs and/or ARBs, statins and fibrates. Regarding the obesity-related indices, the male patients with MetS had higher BMI, WHR, WHtR, LAP, BRI, CI, VAI, BAI, AVI, ABSI and TyG index. Similar results were found in the female patients, except that the female patients with MetS did not have a higher BH than those without MetS.

### Determinants of MetS

Table 2 shows the determinants of MetS in the study patients. In the male patients, after adjusting for age, systolic and diastolic blood pressures, total cholesterol, LDL-C, eGFR and medication use, high BMI (per 1 kg/m^2^; odds ratio [OR] = 1.382; *p* < 0.001), high WHR (per 0.1; OR = 7.027; *p* < 0.001), high WHtR (per 0.1; OR = 11.445; *p* < 0.001), high LAP (per 1; OR = 1.124; *p* < 0.001), high BRI (per 1; OR = 3.213; *p* < 0.001), high CI (per 0.01; OR = 1.175; *p* < 0.001), high VAI (per 1; OR = 9.020; *p* < 0.001), high BAI (per 1; OR = 1.174; *p* < 0.001), high AVI (per 1; OR = 1.560; *p* < 0.001), high ABSI (per 0.01; OR = 3.978; *p* < 0.001), and high TyG index (per 1; OR = 7.370; *p* < 0.001) were significantly associated with MetS. In the female patients, after multivariable adjustments, high BMI (per 1 kg/m^2^; OR = 1.273; *p* < 0.001), high WHR (per 0.1; OR = 3.241; *p* < 0.001), high WHtR (per 0.1; OR = 5.868; *p* < 0.001), high LAP (per 1; OR = 1.118; *p* < 0.001), high BRI (per 1; OR = 2.265; *p* < 0.001), high CI (per 0.01; OR = 1.103; *p* < 0.001), high VAI (per 1; OR = 6.870; *p* < 0.001), high BAI (per 1; OR = 1.141; *p* < 0.001), high AVI (per 1; OR = 1.471; *p* < 0.001), high ABSI (per 0.01; OR = 2.620; *p* < 0.001), and high TyG index (per 1; OR = 8.210; *p* < 0.001) were significantly associated with MetS.

### Interactions among sex and obesity-related indices on MetS

Analysis of the interactions among sex and the obesity-related indices on MetS is also shown in Table 2. The interactions between BMI and sex (*p* = 0.036), WHR and sex (*p* = 0.016), and CI and sex (*p* = 0.026) on MetS were statistically significant. However, there were no significant differences in the interactions between the other indices and sex.

### ROC curve analysis for the obesity-related indices in identifying MetS

**Figure 2:**
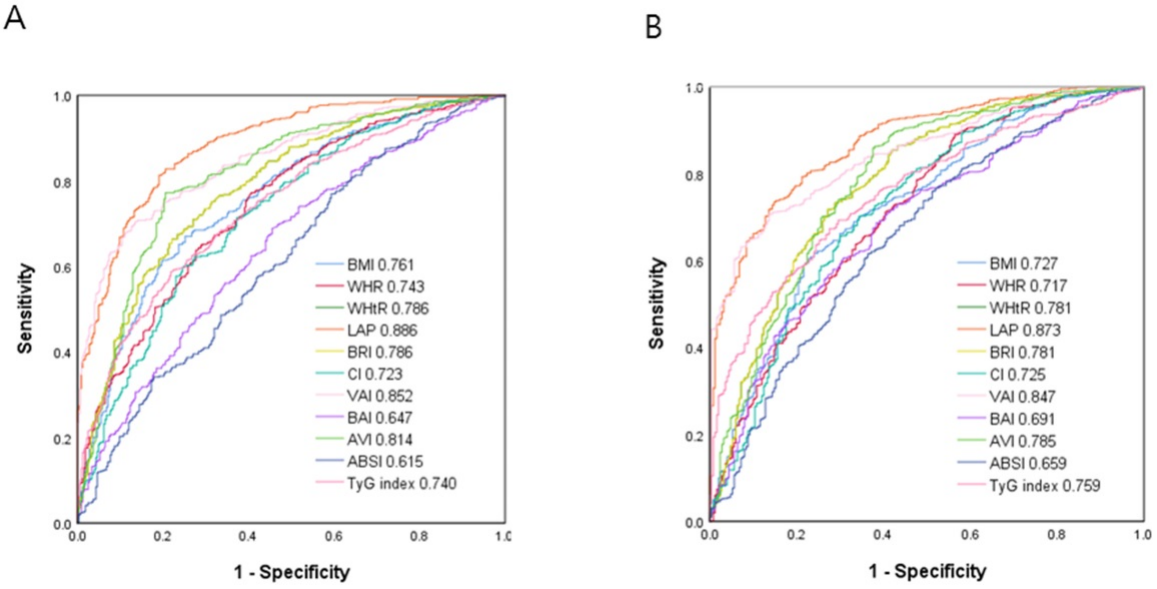
Comparison of the predictive value of 11 obesity-related indices for diagnosis of MetS among (A) males and (B) females.

Figure 2 shows the ROC curves and AUCs of the 11 obesity-related indices in identifying MetS in the male (A) and female (B) patients. In the male patients, LAP had the greatest AUC (AUC = 0.886), followed by VAI (AUC = 0.852), AVI (AUC = 0.814), WHtR and BRI (AUC = 0.786), BMI (AUC = 0.761), WHR (AUC = 0.743), TyG index (AUC = 0.740), CI (AUC = 0.723), BAI (AUC = 0.647) and ABSI (AUC = 0.615). In the female patients, LAP also had the greatest AUC (AUC = 0.873), followed by VAI (AUC = 0.847), AVI (AUC = 0.785), WHtR and BRI (AUC = 0.781), TyG index (AUC = 0.759), BMI (AUC = 0.727), CI (AUC = 0.725), WHR (AUC = 0.717), BAI (AUC = 0.691) and ABSI (AUC = 0.659).

Table 3 and 4 demonstrate the ROC analysis and AUCs, cutoff values, sensitivity, specificity and Youden index of the 11 obesity-related indices for MetS in the male and female patients, respectively.

## Discussion

In this study, we evaluated sex differences in the associations among various obesity-related indices and MetS in patients with type 2 DM. We found that all of the studied obesity-related indices were associated with MetS, and that the interactions between BMI, WHR, and CI and sex on MetS were statistically significant.

The first important finding of this study is that all 11 obesity-related indices (BMI, WHR, WHtR, LAP, BRI, CI, VAI, BAI, AVI, ABSI and TyG index) were associated with MetS in both sex, and that LAP and VAI were the strongest predictors of MetS in both sex. An increase in the prevalence of obesity has been reported in most Asian countries during the last two decades 29. Several studies among different populations have shown that obesity-related indices can be used to predict MetS 11, 13, 14, 30. Xia et al. demonstrated that LAP is a powerful marker of IR among people without diabetes 31. In addition, two other studies found that LAP was an accurate and simple predictor of MetS in a Taiwanese population and children in China 32, 33. We also found that LAP was a powerful predictor of MetS among patients with diabetes. In our study, we found that LAP was also associated with MetS in diabetic population. It is beneficial for LAP to predict the MetS in both diabetic and non-diabetic populations. Several potential mechanisms have been proposed for the association between visceral fat and IR. First, compared to subcutaneous fat, visceral fat has a higher rate of lipolysis. This can lead to higher levels of free fatty acids and subsequently fat accumulation in the liver, and this in turn can induce IR. Second, adipocytokines secreted by visceral fat may induce IR 34. On the other hand, a systematic review indicated that VAI may be a useful predictor of type 2 DM in Asian populations 35. In addition, Baveicy et al. reported that VAI was a valuable predictor of MetS 36. The reason why VAI appears to be a good predictor of MetS may partly be because VAI is highly correlated with WC, TGs and HDL-C, three major components of MetS. Compared to MetS, the advantage of VAI is the reflection of visceral fat distribution. Although computed tomography and magnetic resonance imaging are considered gold standards for the measurement of fat distribution, it takes time and cost to acquire the information. Therefore, non-invasive and inexpensive calculation of VAI can evaluate the adiposity and MetS 11. In addition, VAI has been shown to be inversely associated with adiponectin and highly associated with inflammatory cytokines, all of which can lead to IR 37. Taken together, these findings indicate that LAP and VAI are excellent predictors of MetS among different populations.

The second important finding of this study is that the interactions between BMI, WHR and CI and sex on MetS were statistically significant. Moreover, these associations were more significant in the men than in the women. Numerous studies have investigated the predictive ability of obesity-related indices in identifying MetS in men and women 38-40. However, there is currently no consensus on which obesity-related index is the most powerful predictor. It may depend on age, sex, ethnicity, or diagnostic criteria of MetS 40. Zhang et al. demonstrated that WHtR was the best predictor of MetS in men, while WC and WHtR were equally good predictors in women 41. In addition, Yu et al. reported that WC was a superior index for predicting MetS in men and that WHtR was superior in women 42, and Gu et al. reported that WHtR, BMI and WC had similar predictive ability to identify MetS in men 43. Our findings indicated that the associations of BMI, WHR and CI on MetS were more significant in the men than in the women. One possible explanation is that men are more prone to accumulate fat in the abdomen, whereas women are more prone to accumulate fat in the gluteal region 44. This difference in fat distribution can result in relatively higher WHR and CI in men compared to women. Moreover, sex hormones including estrogen and testosterone have also been associated with body fat distribution 45, 46. Estrogen modulates lipolysis and lipogenesis mainly via estrogen receptor α, and this affects adipose tissue expansion and remodeling. Likewise, androgen modulates lipolysis by suppressing lipoprotein lipase activity and directing fat away from the gluteofemoral region. However, the detailed molecular mechanism by which sex hormones regulate body fat distribution is still unclear 47.

There are several limitations to this study. First, the study included patients with DM regardless of its duration, and the duration of DM can affect the inhomogeneity of the effects of diabetes on MetS. However, DM duration, especially in type 2 diabetes, is a very unreliable subjective statement from patients, and this can lead to bias in studies of diabetes. Nonetheless, the results may help to shed light on the importance of obesity-related indices on MetS in this population. Second, some important variables influencing MetS, such as smoking history, exercise and economic status were lacking. These factors may also be associated with the development of MetS. In addition, all of the participants in this study were recruited in Taiwan, and therefore our results may not be generalizable to other populations. Finally, this study was cross-sectional in design, and therefore we could not confirm causal relationships or long-term clinical outcomes. Further prospective studies are required to evaluate the development and progression of MetS in patients with diabetes.

In conclusion, our results demonstrated significant relationships among obesity-related indices including BMI, WHR, WHtR, LAP, BRI, CI, VAI, BAI, AVI, ABSI and TyG index, and MetS among patients with type 2 DM. Among them, LAP and VAI were the strongest predictors of MetS in both men and women. BMI, WHR and CI were associated with MetS more obviously in the men than in the women. In clinical, these obesity-related indices can be easily calculated by simple anthropometric measurements and laboratory data survey, and then used as predictive tools for MetS.

## Figures and Tables

**Table 1:** Clinical characteristics of the study participants classified by the presence of different sex and MetS

Characteristics	Male (n = 808)			Female (n = 1064)		
	MetS (-) (n = 325)	MetS (+) (n = 483)	*p*	MetS (-) (n = 251)	MetS (+) (n = 813)	*p*
Age (year)	61.0 ± 12.1	62.8 ± 10.8	0.034	60.8 ± 12.6	66.9 ± 12.1	< 0.001
Systolic BP (mmHg)	126.1 ± 15.2	138.8 ± 18.8	< 0.001	124.2 ± 14.5	139.5 ± 18.7	< 0.001
Diastolic BP (mmHg)	75.6 ± 10.6	81.5 ± 11.5	< 0.001	73.0 ± 8.9	78.2 ± 11.3	< 0.001
Body height (cm)	165.1 ± 6.5	166.4 ± 6.0	0.003	153.9 ± 5.6	153.7 ± 5.5	0.530
Body weight (Kg)	65.1 ± 9.4	74.2 ± 10.1	< 0.001	56.8 ± 8.4	63.3 ± 9.2	< 0.001
Waist circumference (cm)	86.1 ± 7.5	95.4 ± 8.1	< 0.001	80.8 ± 9.0	90.4 ± 8.6	< 0.001
Hip circumference (cm)	95.3 ± 6.0	100.5 ± 7.4	< 0.001	94.5 ± 7.1	99.8 ± 7.7	< 0.001
**Laboratory parameters**						
Fasting glucose (mg/dL)	148.6 ± 54.7	146.3 ± 52.3	0.546	144.6 ± 50.4	151.1 ± 51.0	0.077
HbA_1c_ (%)	7.6 ± 1.9	7.7 ± 1.6	0.830	7.5 ± 1.6	7.7 ± 1.6	0.056
Triglyceride (mg/dL)	91 (68.5-120)	158 (112-214)	< 0.001	92 (68-135)	146 (106-196)	< 0.001
Total cholesterol (mg/dL)	178.0 ± 35.6	181.6 ± 40.8	0.204	189.1 ± 30.4	189.9 ± 39.4	0.769
HDL-cholesterol (mg/dL)	51.5 ± 11.4	41.9 ± 9.8	< 0.001	62.9 ± 12.1	49.5 ± 12.2	< 0.001
LDL-cholesterol (mg/dL)	103.8 ± 28.5	103.5 ± 28.4	0.894	102.6 ± 25.0	105.7 ± 28.9	0.099
eGFR (mL/min/1.73 m^2^)	73.3 ± 17.7	65.5 ± 20.3	< 0.001	76.2 ± 19.8	65.4 ± 20.7	< 0.001
**Medications**						
Oral anti-diabetic drugs (%)	85.2	90.9	0.013	85.1	93.1	< 0.001
Insulin (%)	37.3	41.2	0.277	37.5	41.5	0.261
ACEI and/or ARB (%)	57.4	80.2	< 0.001	52.0	82.5	< 0.001
Statins use (%)	48.1	58.0	0.006	56.0	66.1	0.004
Fibrate use (%)	4.6	26.6	< 0.001	6.0	18.1	< 0.001
**Obesity-related indices**						
BMI (kg/m^2^)	23.8 ± 2.8	26.7 ± 3.2	< 0.001	23.9 ± 3.3	26.7 ± 3.6	< 0.001
WHR	0.90 ± 0.05	0.95 ± 0.05	< 0.001	0.86 ± 0.07	0.91 ± 0.06	< 0.001
WHtR	0.52 ± 0.05	0.57 ± 0.05	< 0.001	0.53 ± 0.06	0.59 ± 0.0	< 0.001
LAP	23.6 ± 13.3	64.6 ± 57.2	< 0.001	24.6 ± 13.6	60.9 ± 39.1	< 0.001
BRI	3.8 ± 0.9	4.9 ± 1.1	< 0.001	3.9 ± 1.3	5.2 ± 1.4	< 0.001
CI	1.26 ± 0.06	1.31 ± 0.06	< 0.001	1.22 ± 0.09	1.30 ± 0.09	< 0.001
VAI	1.1 ± 0.6	2.9 ± 3.3	< 0.001	1.3 ± 0.7	3.3 ± 3.1	< 0.001
BAI	27.0 ± 3.1	28.9 ± 4.0	< 0.001	31.6 ± 4.1	34.5 ± 4.7	< 0.001
AVI	15.0 ± 2.6	18.4 ± 3.2	< 0.001	13.4 ± 3.0	16.6 ± 4.1	< 0.001
ABSI	0.081 ± 0.004	0.083 ± 0.004	< 0.001	0.079 ± 0.006	0.082 ± 0.006	< 0.001
TyG index	8.8 ± 0.5	9.3 ± 0.7	< 0.001	8.7 ± 0.5	9.3 ± 0.7	< 0.001
**Components of MetS**						
Central obesity (%)	20.4	77.0	< 0.001	42.2	89.8	< 0.001
High BP (%)	30.8	82.6	< 0.001	21.2	83.4	< 0.001
Low HDL-cholesterol (%)	6.8	51.3	< 0.001	8.0	58.1	< 0.001
High triglyceride (%)	6.5	55.7	< 0.001	2.0	48.2	< 0.001

Abbreviations: MetS, metabolic syndrome; BP, blood pressure; HbA_1c_, glycated hemoglobin A_1c_; HDL, high-density lipoprotein; LDL, low-density lipoprotein; eGFR, estimated glomerular filtration rate; ACEI, angiotensin converting enzyme inhibitor; ARB, angiotensin II receptor blocker; BMI, body mass index; WHR, waist-hip ratio; WHtR, waist-to-height ratio; LAP, lipid accumulation product; BRI, body roundness index; CI, conicity index; VAI, visceral adiposity index; BAI, body adiposity index; AVI, abdominal volume index; ABSI, a body shape index; TyG index, triglyceride glucose index.

**Table 2:** Association of obesity-related indices with MetS using multivariable logistic regression analysis

Obesity-related indices	Multivariate						Interaction *p*
	Male (n = 808)			Female (n = 1064)			
	OR	95% confidence interval	*p*	OR	95% confidence interval	*p*	
BMI (per 1 kg/m^2^)	1.382	1.294-1.476	< 0.001	1.273	1.201-1.350	< 0.001	0.036
WHR (per 0.1)	7.027	4.685-10.540	< 0.001	3.241	2.371-4.430	< 0.001	0.016
WHtR (per 0.1)	11.445	7.255-18.052	< 0.001	5.868	4.101-8.397	< 0.001	0.111
LAP (per 1)	1.124	1.103-1.145	< 0.001	1.118	1.098-1.139	< 0.001	0.666
BRI (per 1)	3.213	2.572-4.012	< 0.001	2.265	1.907-2.690	< 0.001	0.078
CI (per 0.01)	1.175	1.136-1.215	< 0.001	1.103	1.077-1.129	< 0.001	0.026
VAI (per 1)	9.020	6.294-12.926	< 0.001	6.870	4.869-9.692	< 0.001	0.096
BAI (per 1)	1.174	1.113-1.237	< 0.001	1.141	1.093-1.190	< 0.001	0.761
AVI (per 1)	1.560	1.442-1.688	< 0.001	1.471	1.364-1.587	< 0.001	0.470
ABSI (per 0.01)	3.978	2.485-6.370	< 0.001	2.620	1.870-3.671	< 0.001	0.583
TyG index (per 1)	7.370	5.012-10.838	< 0.001	8.210	5.663-11.902	< 0.001	0.745

Values expressed as odds ratio (OR) and 95% confidence interval. Abbreviations are the same as in Table 1. Covariates in the multivariable model included age, systolic and diastolic blood pressures, total cholesterol, LDL-cholesterol, eGFR and medications use.

**Table 3:** Area under curve (AUC), cutoff value, Youden index, sensitivity and specificity of 11 obesity-related indices in male patients

Obesity-related indices	AUC (95% Confidence Interval)	Cutoff Value	Sensitivity (%)	Specificity (%)	Youden Index
BMI (kg/m^2^)	0.761 (0.728-0.795)*	25.021	69.2	69.2	0.384
WHR	0.743 (0.709-0.778)*	0.929	67.3	67.4	0.347
WHtR	0.786 (0.754-0.818)*	0.542	71.8	71.7	0.435
LAP	0.886 (0.863-0.909)*	33.236	81.4	80.9	0.623
BRI	0.786 (0.754-0.818)*	4.187	71.8	71.6	0.434
CI	0.723 (0.687-0.759)*	1.286	65.6	65.6	0.311
VAI	0.852 (0.826-0.877)*	1.509	76.2	76.3	0.525
BAI	0.647 (0.609-0.685)*	27.563	60.5	60.0	0.205
AVI	0.814 (0.784-0.845)*	16.240	77.4	77.2	0.564
ABSI	0.615 (0.576-0.655)*	0.820	57.3	58.2	0.155
TyG index	0.740 (0.706-0.774)*	8.988	67.7	67.1	0.348

*p < 0.001. Abbreviations are the same as in Table 1.

**Table 4:** Area under curve (AUC), cutoff value, Youden index, sensitivity and specificity of 11 obesity-related indices in female patients

Obesity-related indices	AUC (95% Confidence Interval)	Cutoff Value	Sensitivity (%)	Specificity (%)	Youden Index
BMI (kg/m^2^)	0.727 (0.691-0.763)*	24.969	67.5	67.6	0.351
WHR	0.717 (0.679-0.754)*	0.874	65.3	64.8	0.301
WHtR	0.781 (0.746-0.815)*	0.552	71.9	72.0	0.439
LAP	0.873 (0.850-0.896)*	33.428	79.3	78.8	0.581
BRI	0.781 (0.746-0.815)*	4.392	71.9	72.0	0.439
CI	0.725 (0.687-0.762)*	1.251	67.0	67.2	0.342
VAI	0.847 (0.823-0.871)*	1.643	76.1	76.0	0.521
BAI	0.691 (0.654-0.728)*	32.446	63.4	63.2	0.266
AVI	0.785 (0.750-0.820)*	14.527	72.3	71.2	0.435
ABSI	0.659 (0.619-0.698)*	0.794	62.4	62.0	0.244
TyG index	0.759 (0.727-0.790)*	8.960	69.3	70.0	0.393

*p < 0.001. Abbreviations are the same as in Table 1.
